# Plant growth promoting fungi suppress the infection with powdery mildew in wheat plants

**DOI:** 10.3389/fpls.2026.1963472

**Published:** 2026-09-23

**Authors:** Faisal Ay Alzahrani, Mohsen M. Elsharkawy

**Affiliations:** 1 Department of Chemistry, College of Sciences and Arts, King Abdulaziz University, Rabigh, Saudi Arabia; 2 Department of Agricultural Botany, Faculty of Agriculture, Kafrelsheikh University, Kafr Elsheikh, Egypt

**Keywords:** *Blumeria graminis* f. sp. *tritici*, defense enzymes, defense genes, induced systemic resistance, plant growth-promoting fungi, wheat

## Abstract

**Introduction:**

*Triticum aestivum* L. (wheat) is severely affected by powdery mildew in Egypt and worldwide, which is caused by the fungus Blumeria graminis f. sp. tritici (Bgt).

**Methods:**

The effectiveness of *Penicillium simplicissimum* GP17-2, *Fusarium equiseti* GF18.3 and GF19.1, *Trichoderma asperellum* SKT-1, *Phoma* sp. GS7.3, GS8.1 and GS8.3, and *Coniothyrium aleuritis* GU23.3 on controlling powdery mildew was evaluated under greenhouse conditions.

**Results and Discussion:**

The PGPF (plant growth-promoting fungi) strains showed a reduction in symptoms and disease progression. Wheat plants treated with PGPF strains showed increased activities of catalase and peroxidase enzymes as well as transcriptions of the *PR1-PR5* and *PR10* genes at 1- and 2-days post-inoculation. The results lead us to believe that PGPF strains might be a promising environmentally friendly method to enhance plant growth and control wheat powdery mildew.

## Introduction

The obligate biotrophic fungus *Blumeria graminis* f. sp. *tritici* has been identified as a serious pathogen (Draz et al., 2019). Powdery mildew is a substantial constraint on wheat production. The pathogen infects the wheat plant’s stems, leaves, and heads, along with other aerial parts (Hong et al., 2018). The fungus adversely impacts grain yield components, reduces the leaf assimilation index, and decreases the capacity of photosynthesis on leaves. Infection during the tillering, booting, and stem elongation stages has a significant impact on yield, especially if it happens early (Draz et al., 2019). This leads to a lower kernel weight and, eventually, a lower yield (Alam et al., 2013). Wheat powdery mildew infections may cause losses in grain production (up to 45%). These losses are particularly severe when the wheat flag leaf gets infected before or during flowering. Powdery mildew has caused documented production losses (up to 26.7%) in Egypt’s wheat cultivars in recent decades, an occurrence that is associated with climatic changes and genetic composition of the pathogen and the host (Draz et al., 2019). The fungus is constantly evolving in terms of its pathotype, and new races are being produced via genetic recombination and mutation (Asad et al., 2014).

The main approaches used to control wheat powdery mildew are synthetic fungicides and resistant cultivars. Therefore, it is crucial to regularly update resistance genes and conduct continuous pathogen monitoring (Draz et al., 2023; Ghoniem et al., 2026). Fungicide application is still the most recommended and practicable management strategy, but it comes with a lot of concerns, including high prices, environmental hazards, and the possibility of developing resistant to fungicides. These difficulties demonstrate the need of integrated management strategies for long-term efficacy against the disease, including biological control, durable resistance, and appropriate fungicide practices. The assessment of non-chemical, biologically based methods for reducing wheat diseases, such as powdery mildew, in greenhouse and field conditions has been encouraged by increasing interest in the sustainable management of diseases. Biocontrol is attracting the attention of experts since it offers a safe and environmentally friendly approach to manage plant diseases (Lugtenberg and Kamilova, 2009).

Plants have various defense systems that may be rapidly activated in response to biotic and abiotic stresses (Choudhary et al., 2016). Plant rhizosphere microorganisms may enhance plant resistance. One of the most encouraging developments in the field of plant resistance is the use of specific formulations of plant growth-promoting fungi (PGPF). For instance, *Coniothyrium aleuritis* and *Penicillium simplicissimum* enhanced the defense of all plant parts against a variety of viral diseases (Elsharkawy et al., 2012, 2024). The PGPF are directly or indirectly contributing to plant growth promotion by secreting various regulatory and antagonistic compounds in the rhizosphere (Glick, 2012; Elsharkawy et al., 2024). Plants contain a wide range of active defense responses to pathogen invasions, including cell wall strengthening and development of antimicrobial bioactive chemicals such as pathogenesis-related proteins (Fiorin et al., 2018). The main mechanisms for PGPF biocontrol activities include antimicrobial metabolite synthesis, competition for resources, and ISR (induced systemic resistance) (Walters et al., 2013). PGPF may usually produce defense substances such as ophiosetin, harzianic acid, the antifungal chitinases, and HCN that are induced by the attack of plant pathogens (Bhattacharyya and Jha, 2012). Rhizofungi may lower the plant pathogenic activity not only via microbial antagonism but also by activating ISR (Zamioudis et al., 2015). A network of consistent signaling pathways controls induced defense systems of plants against pathogens. The primary focuses of the network are the plant signal molecules–jasmonic acid (JA), SA, ethylene (ET), and potentially nitric oxide (NO) (Annapurna et al., 2013). The results of recent research indicate that beneficial microbes trigger early stimulation responses in plants including the upregulation of genes related to pathogenesis (PR genes such as *PR1, PR2, PR3, PR4, PR5* and *PR10* (Wang et al., 2020). Therefore, PGPF may be employed as an alternative option to common fungicides for improving wheat yield and ecologically safe protection against wheat diseases. However, the precision mechanisms by which they work remain largely unknown. Finding additional information on this subject was the purpose of this research. The present research was designed to evaluate the potentiality of PGPF strains to produce ISR in wheat plants against fungal powdery mildew pathogen, *B. graminis* f. sp. *tritici*, and to monitor the defense-related genes *PR1, PR2, PR3, PR4, PR5* and *PR10* that are triggered more actively in pathogen-infected systemically resistant plants.

## Materials and methods

### Barley grain inoculum preparation

PGPF strains were obtained from Gifu University, Japan. Stock cultures of PGPF were grown on slant cultures of MEA (2%) cultured at 25 °C and stored at 4 °C. Barley grains (100 g) were autoclaved together with 100 ml of distilled water. Agar discs (5-mm diameter) from the new cultures of *Penicillium simplicissimum* GP17-2, *Fusarium equiseti* GF18.3 and GF19.1, *Trichoderma asperellum* SKT-1, *Phoma* sp. GS7.3, GS8.1 and GS8.3, and *Coniothyrium aleuritis* GU23.3 were inoculated after the barley grains had cooled. Completely colonized BGI of PGPF were air-dried at 27 °C. Dried BGI were grinded into 2-mm particles. BGI of PGPF strains were mixed with clay soil (2% W/W) and sterilized barley grains were used for the control. Sakha-61 cultivar was grown in plastic pots (10 cm in diameter, 10 grains/pot). Pots were manually irrigated every three days with tap water and fertilized as recommended. There were three independent repetitions of each treatment.

### Pathogen inoculation and disease assessment

Wheat grains were obtained from PPRI, Agricultural Research Center, Egypt. Plants of reference treatment were treated with the liquid fungicide Tebuconazole at a concentration of 25% (0.25 ml/L, foliar spray at 1 day before pathogen inoculation). The Bgt inoculum was collected from wheat plants that were naturally infected and had newly formed powdery mildew lesions with active sporulation (Esmail et al., 2026). One day before inoculation, the infected plants were shaken to eliminate older conidia, allowing for the collection of highly viable and fresh conidia. Inoculation was performed using freshly generated conidia. The susceptible wheat cultivar Sakha-61 was used for inoculum propagation according to Browder (1972). A uniform distribution of conidia (70 to 80 conidia/mm² of leaf surface) was performed by carefully brushing over 1-week-old seedlings. The plants were placed in the dark for 24 h at 18 °C and then moved to the greenhouse (temperature at 18 °C, 100% relative humidity and 15/9 h day/night photoperiod). Plant height and shoot fresh weight were evaluated at 4-week-old plants.

Seedling reactions were assessed at two weeks post-inoculation using the 0–4 infection type rating method as outlined by Shi et al. (1987). Types 0–2 were classified as R (resistant), whereas types 3 and 4 were classified as S (susceptible). The IP (incubation period) and LP (latent period) were measured according to the protocol described by Parlevliet (1975). The severity of the mildew was determined as explained by Sharma and Duveiller (2007). The efficacy (%) of the PGPF strains tested against wheat powdery mildew at the seedling stage was determined using the technique proposed by Rewal and Jhooty (1985).


$$\text{Efficacy }(\%)=\frac{\text{Disease severity in control }-\text{Disease severity in treatment}}{\text{Disease severity in control}}\times 100$$

### Antioxidant enzymes assay

Enzyme analyses were conducted using freshly harvested wheat leaves (0.5 g) which were homogenized in polyvinylpyrrolidone (7.5%) and Tris buffer (3 mL, 50 mM, pH 7.8, contained EDTA-Na_2–_1 mM). The mixture was centrifuged for 20 min at 12,000 rpm (Elsharkawy et al., 2022). The spectrophotometer (UV-160A, Shimadzu, Japan) was utilized for all experiments at 25 °C. The activity of catalase was assessed (Aebi, 1984). The absorbance at 240 nm was measured every 30 s for 3 min. The peroxidase activity was measured in the extracted crude enzyme following the method of Hammerschmidt et al. (1982). The absorbance at 470 nm was measured every 30 s for 3 min.

### Colonization of wheat roots with PGPF

Wheat roots were tested for PGPF colonization at 7-week-old plants. Ten roots from randomly selected plants were collected, washed with SDW (sterile distilled water) to eliminate any residual soil, immersed in 70% ethanol to eradicate any external contamination, washed again three times with SDW, then blotted dry and placed on PDA plates. The colonies were counted from 100 root segments independently for each biological replicate using the basic morphology of each isolate as a guide (Elsharkawy et al., 2024).

### qRT-PCR analysis

In order to extract RNA, samples of wheat were taken from the first leaf. Total RNA was extracted from 100 mg of wheat leaves at 1, and 2 days post-inoculation (DPI) for each treatment. A kit (#K0731, Thermo Scientific, Fermentas) was used for RNA purification. Using agarose gel electrophoresis and Nano SPECTROstar, the RNA’s content, purity, and integrity were checked. The kit (#EP0451, Thermo Scientific, Fermentas) for reverse transcription was used for the procedure. The transcription patterns of the wheat genes (*PR1, PR2, PR3, PR4, PR5* and *PR10*) were identified by qRT-PCR amplification of the produced cDNA using specified primers. A real-time cycler was used to amplify the transcripts of all genes (Desmond et al., 2006). The *β-tubulin* reference gene was utilized to normalize gene expressions (**Table 1**). There was a total of three biological and technical replicates utilized for every treatment. The relative expression levels were determined by using the approach developed by Livak and Schmittgen (2001).

**Table 1:** The nucleotide sequences of primers used in this study.

Primer name	Forward primer (5′……3′)	Reverse primer (5′……3′)
*PR1*	CTGGAGCACGAAGCTGCAG	CGAGTGCTGGAGCTTGCAGT
*PR2*	CTCGACATCGGTAACGACCAG	GCGGCGATGTACTTGATGTTC
*PR3*	AGAGATAAGCAAGGCCACGTC	GGTTGCTCACCAGGTCCTTC
*PR4*	CGAGGATCGTGGACCAGTG	GTCGACGAACTGGTAGTTGACG
*PR5*	ACAGCTACGCCAAGGACGAC	CGCGTCCTAATCTAAGGGCAG
*PR10*	TTAAACCAGCACGAGAAACATCAG	ATCCTCCCTCGATTATTCTCACG
*β-tubulin*	GCCATGTTCAGGAGGAAGG	CTCGGTGAACTCCATCTCGT

### Statistical analysis

There were three independent trials of every experiment. ANOVA (Analysis of variance) *post hoc* tests were conducted. The results were shown using the means for every set of replicates. The Excel Tokei statistical program was used to assess the statistical significance of differences across means by employing the LSD (Least Significant Difference) at *p < 0.05*, while *post-hoc* Steel-Dwass test was used for disease assessment.

## Results

### Induced resistance against powdery mildew by PGPF

Wheat plants treated with PGPF strains *P. simplicissimum* GP17-2, *F. equiseti* GF18.3 and GF19.1, *T. asperellum* SKT-1, *Phoma* sp. GS7.3, GS8.1 and GS8.3, and *C. aleuritis* GU23.3 showed significant resistance against powdery mildew (**Table 2**). The effects of the PGPF on the development of wheat powdery mildew disease were measured by changes in IT, IP, and LP (**Table 2**). The greatest inhibitory effect was observed with *P. simplicissimum* GP17-2, which had a substantially higher LP of 15 days and an IP value of 10 days than other treatments, with a low infection type of 1. *F. equiseti* GF18.3 and GF19.1, *Phoma* sp. GS7.3 and *C. aleuritis* GU23.3 maintained IP and LP values of 10 and 14 days, respectively, with a low IT of 1. *T. asperellum* SKT-1 and *Phoma* sp. GS8.1 both resulted in low infection types of 2, with IP of 9 days and LP of 12 days compared to the control (**Table 2**).

**Table 2:** The potential of the examined PGPF strains on the wheat powdery mildew disease development during the seedling stage using the infection type (IT), incubation period (IP) in days, and latent period (LP) in days.

Treatment	IT	IP	LP
Control	4^a^*	7 ± 0.17^c^	9 ± 0.16^e^
Tebuconazole	0	–	–
*Penicillium simplicissimum* GP17-2	1^c^	10 ± 0.22^a^	15 ± 0.22^a^
*Trichoderma asperellum* SKT-1	2^b^	9 ± 0.20^b^	12 ± 0.21^c^
*Fusarium equiseti* GF18.3	1^c^	10 ± 0.25^a^	14 ± 0.20^b^
*Fusarium equiseti* GF19.1	1^c^	10 ± 0.21^a^	14 ± 0.25^b^
*Phoma* sp. GS7.3	1^c^	10 ± 0.19^a^	14 ± 0.20^b^
*Phoma* sp. GS8.1	2^b^	9 ± 0.21^b^	12 ± 0.21^c^
*Phoma* sp. GS8.3	2^b^	9 ± 0.22^b^	12 ± 0.24^c^
*Coniothyrium aleuritis* GU23.3	1^c^	10 ± 0.23^a^	14 ± 0.22^b^

*There is no significant difference between means that are followed by the same letter in each column. Values are means ± standard error (SE).

The PGPF treatments were far more effective in reducing powdery mildew symptoms than the check control (**Figure 1**). Plants treated with *P. simplicissimum* GP17–2 inoculation consistently displayed less severe symptoms than other PGPF strains (**Figure 1**). *P. simplicissimum* GP17–2 treatment showed a considerable increase in the effectiveness % compared with the control treatment (**Figure 2**) (. *T. asperellum* SKT-1 considerably decreased powdery mildew severity but with less order. Disease severity was significantly different in all treatments compared to the untreated control (*p < 0.05*). Generally, lesion size was lower in pre-treated wheat plants compared to control plants.

**Figure 1:**
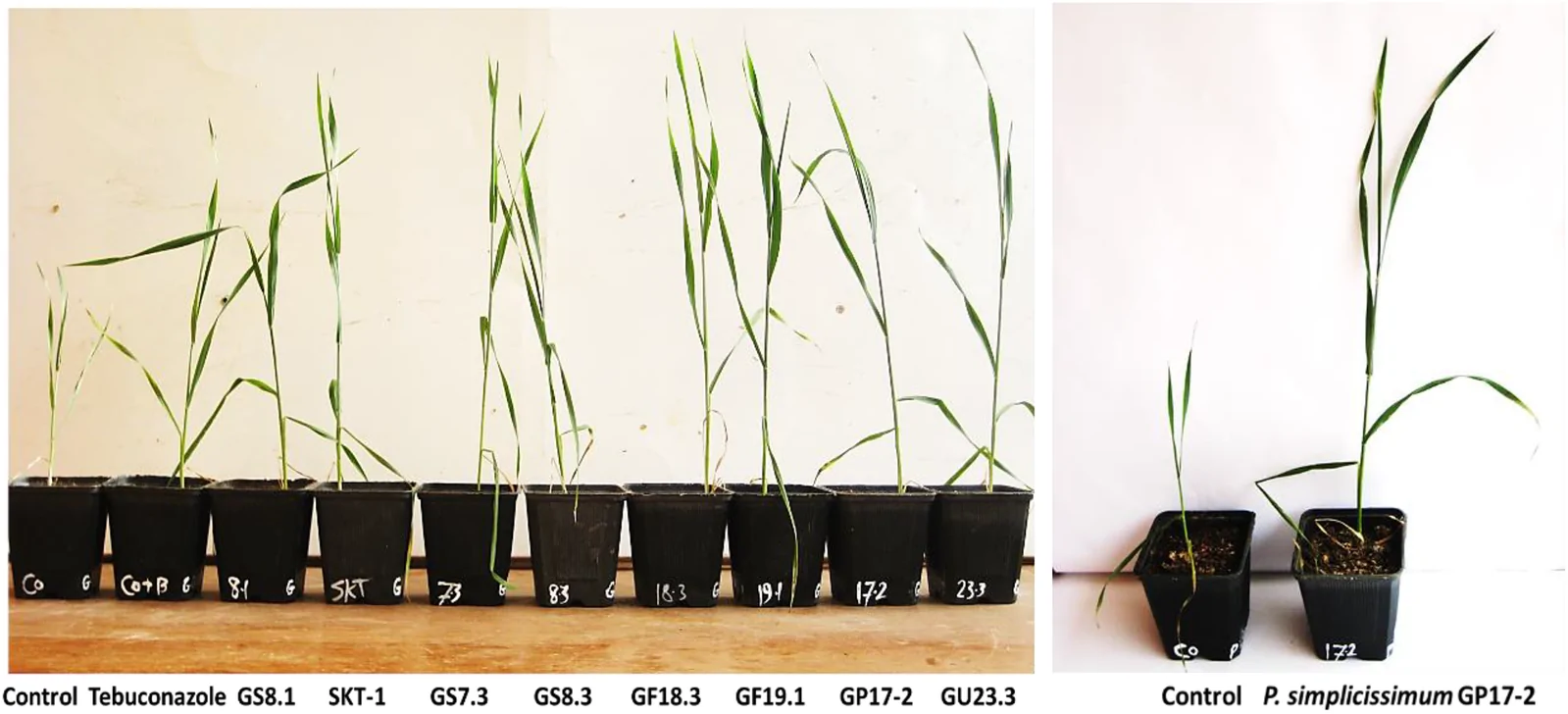
Effect of PGPF application on wheat growth and development at 25-day-old seedlings.

**Figure 2:**
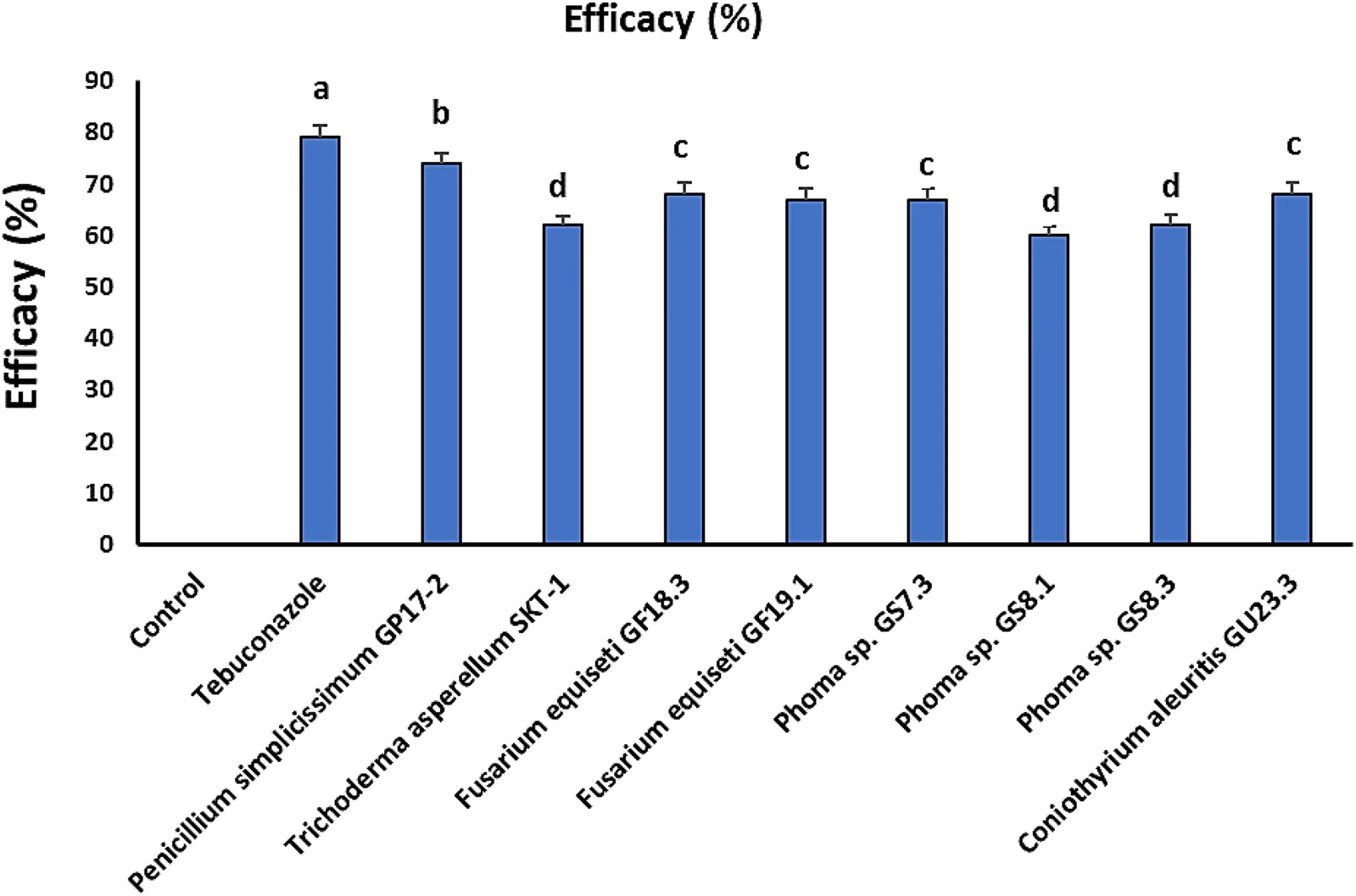
The efficacy percentage of PGPF treatments against wheat powdery mildew. Values are means ± standard error (SE). Different letters indicate statistical significance between treatments according to the *Post-hoc* Steel-Dwass test.

### Stimulation of wheat growth by PGPF strains

Among the PGPF strains applied to soil, *C. aleuritis* GU23.3 and *F. equiseti* GF18.3 significantly improved plant height (32.4 cm) and shoot fresh weight (1.7 g) compared with the control. *Phoma* sp. GS8.3 treatment resulted in the lowest enhancement in plant development recording 30 cm of plant height and 1.5 g of shoot fresh weight (**Table 3**).

**Table 3:** The potential of the examined PGPF strains on the growth parameters of wheat plants at 4-week-old.

Treatment	Plant height (cm)	Shoot fresh weight (g)
Control	19.1 ± 0.27^d^	0.7 ± 0.06^d^
Tebuconazole	26.9 ± 0.36^c^	1.1 ± 0.05^c^
*Penicillium simplicissimum* GP17-2	32.3 ± 0.28^a^	1.7 ± 0.06^a^
*Trichoderma asperellum* SKT-1	30.1 ± 0.30^b^	1.5 ± 0.05^b^
*Fusarium equiseti* GF18.3	32.4 ± 0.39^a^	1.7 ± 0.04^a^
*Fusarium equiseti* GF19.1	32.3 ± 0.31^a^	1.7 ± 0.06^a^
*Phoma* sp. GS7.3	32.1 ± 0.29^a^	1.7 ± 0.05^a^
*Phoma* sp. GS8.1	30.2 ± 0.33^b^	1.5 ± 0.04^b^
*Phoma* sp. GS8.3	30.0 ± 0.28^b^	1.5 ± 0.05^b^
*Coniothyrium aleuritis* GU23.3	32.4 ± 0.33^a^	1.7 ± 0.06^a^

*There is no significant difference between means that are followed by the same letter in each column. Values are means ± standard error (SE).

### Re-isolation frequency of PGPF

Our results show that 89%-95% of wheat plants had substantial frequencies of PGPF strains at 7-week-old plants. *P. simplicissimum* GP17–2 treatment resulted in the highest frequency from wheat roots (95%), while *Phoma* sp. GS8.1 exhibited the lowest frequency percentage (89%) (**Table 4**).

**Table 4:** Re-isolation frequencies of PGPF strains from wheat roots at 7-week-old plants.

Treatment	Re-isolation frequency (%)
Control	0^d^
*Penicillium simplicissimum* GP17-2	95 ± 1.2^a*^
*Trichoderma asperellum* SKT-1	92 ± 0.9^b^
*Fusarium equiseti* GF18.3	92 ± 1.0^b^
*Fusarium equiseti* GF19.1	92 ± 0.9^b^
*Phoma* sp. GS7.3	90 ± 0.8^c^
*Phoma* sp. GS8.1	89 ± 1.0^c^
*Phoma* sp. GS8.3	90 ± 0.9^c^
*Coniothyrium aleuritis* GU23.3	92 ± 1.0^b^

*There is no significant difference between means that are followed by the same letter in each column. Values are means ± standard error (SE).

### Assessment of defense enzymes

**Figures 3** and **4** showed the results of the catalase and peroxidase activities. The activities of peroxidase and catalase in wheat leaves infected with Bgt were significantly influenced by treatments with PGPF strains (**Figures 3** and **4**). All PGPF treatments markedly improved the levels of catalase and peroxidase compared to the untreated control. *P. simplicissimum* GP17–2 demonstrated the highest induction of antioxidant enzyme activity, with CAT at 89.05 mM H_2_O_2_ g^-1^ FW min^-1^ and peroxidase at 0.055 mM H_2_O_2_ g^-1^ FW min^-1^ (**Figures 3** and **4**). This was followed by *Phoma* sp. GS7.3 and *C. aleuritis* GU23.3, which also resulted in elevated activities, indicating very active metabolisms in treated plants. However, *Phoma* sp. GS8.1 induced the lowest enzyme activities among the PGPF treatments. Moderate amounts of CAT (64.1) and POX (0.03) were produced by Tebuconazole but were still much higher than in the control group.

**Figure 3:**
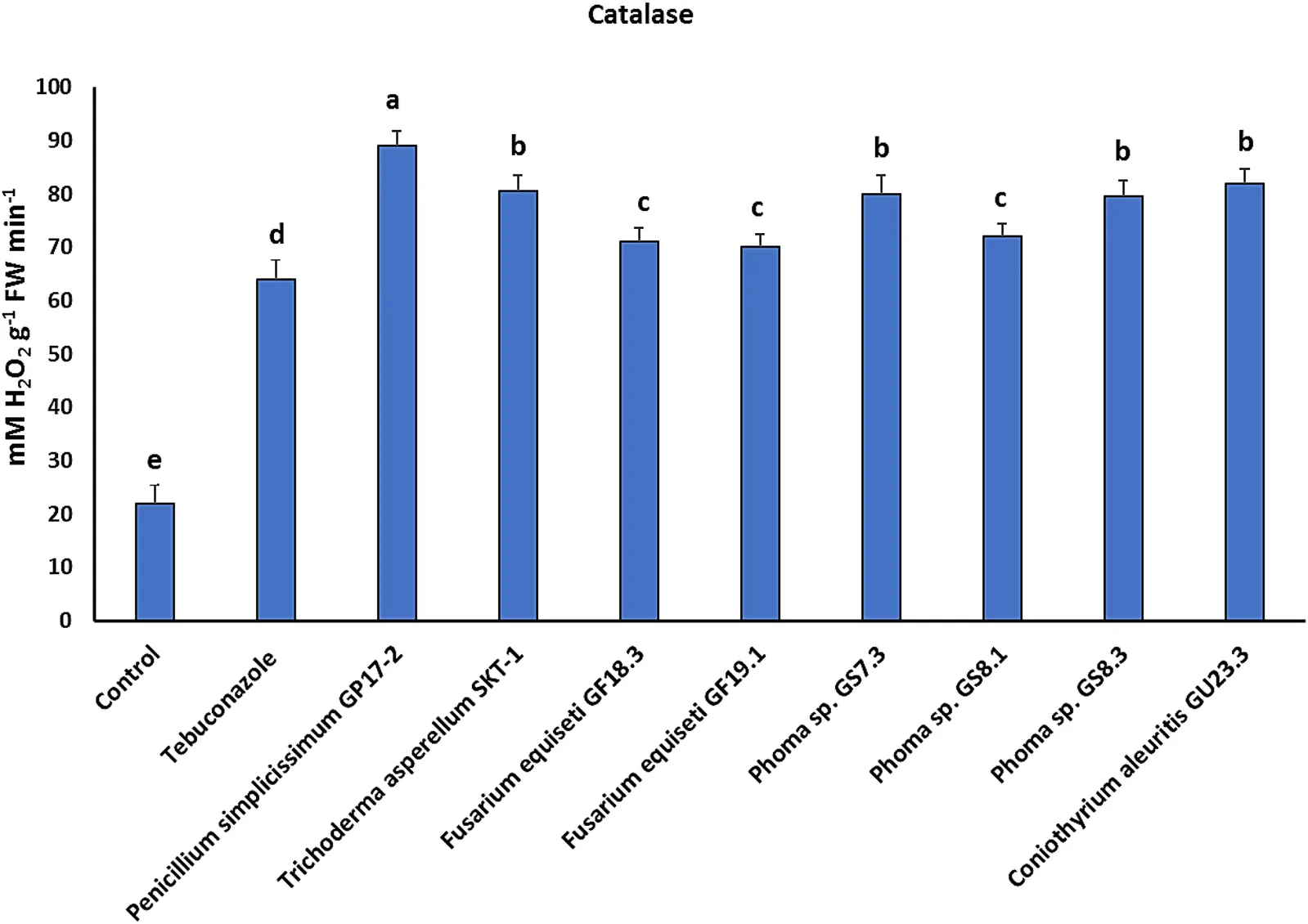
Effect of PGPF treatments on the activity of catalase in *B. graminis* f. sp. *tritici-*infected wheat plants. Values are means ± standard error (SE). Different letters indicate statistical significance between treatments at *p < 0.05*.

**Figure 4:**
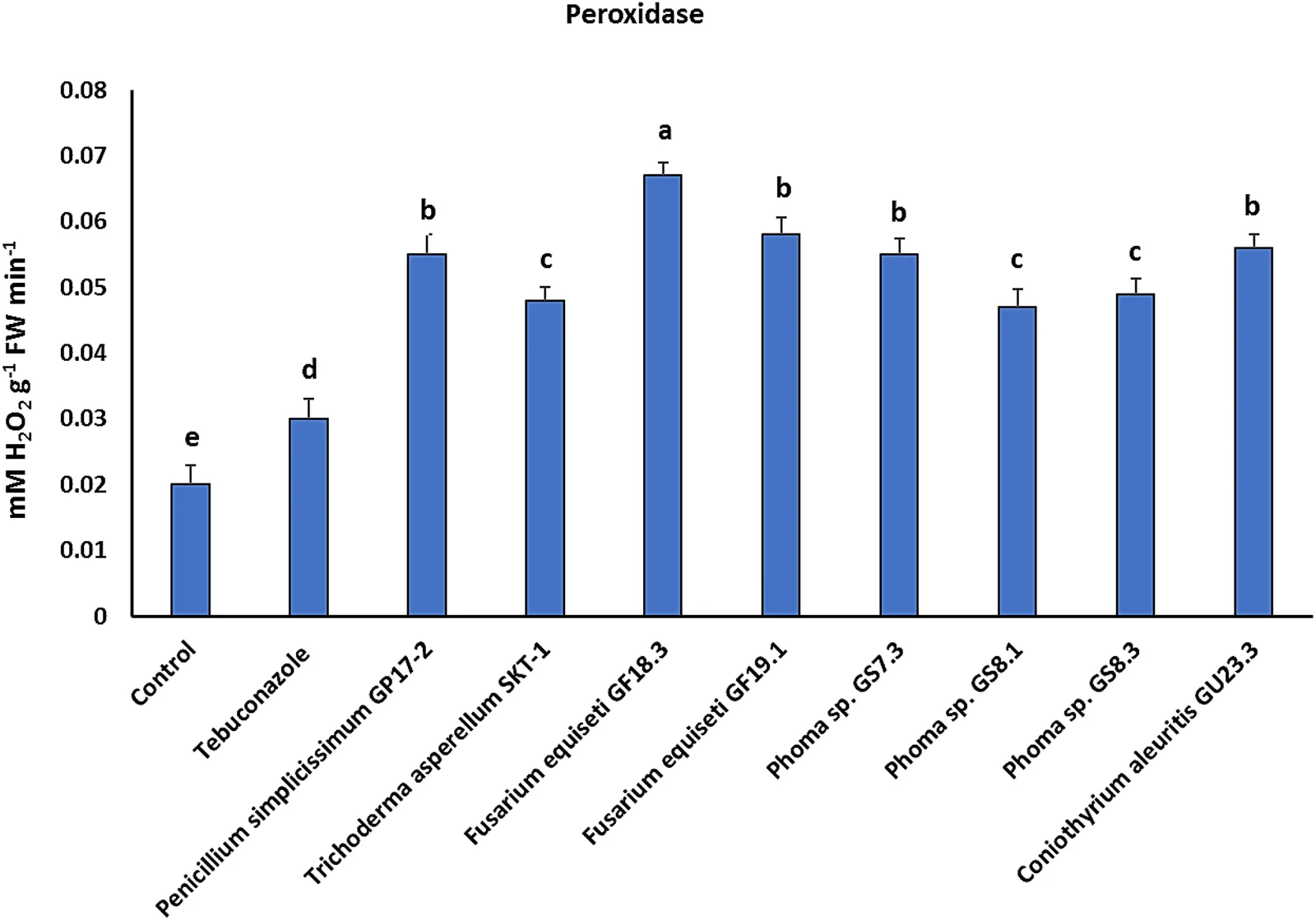
Effect of PGPF treatments on the activity of peroxidase in *B. graminis* f. sp. *tritici-*infected wheat plants. Values are means ± standard error (SE). Different letters indicate statistical significance between treatments at *p < 0.05*.

### Relative expression of defense genes

The involvement of certain defense genes in response to Bgt using the qRT-PCR method was explored. Pathogenesis-related genes (*PR1-PR5* and *PR10*) transcription levels were examined at time intervals of 1 and 2 DPI (**Figures 5**–**10**). The results showed a high accumulation of *PR1–5* and *PR10* in the wheat plants inoculated with PGPF compared to noninoculated groups. QRT-PCR revealed that a barley grain inoculum of *P. simplicissimum* GP17–2 increased the expression of all defense genes. However, treatment with *F. equiseti* GF18.3 highly stimulated the expression of the *PR2*, *PR5* and *PR10*, relative to other treatments (**Figures 6**, **8** and **10**).

**Figure 5:**
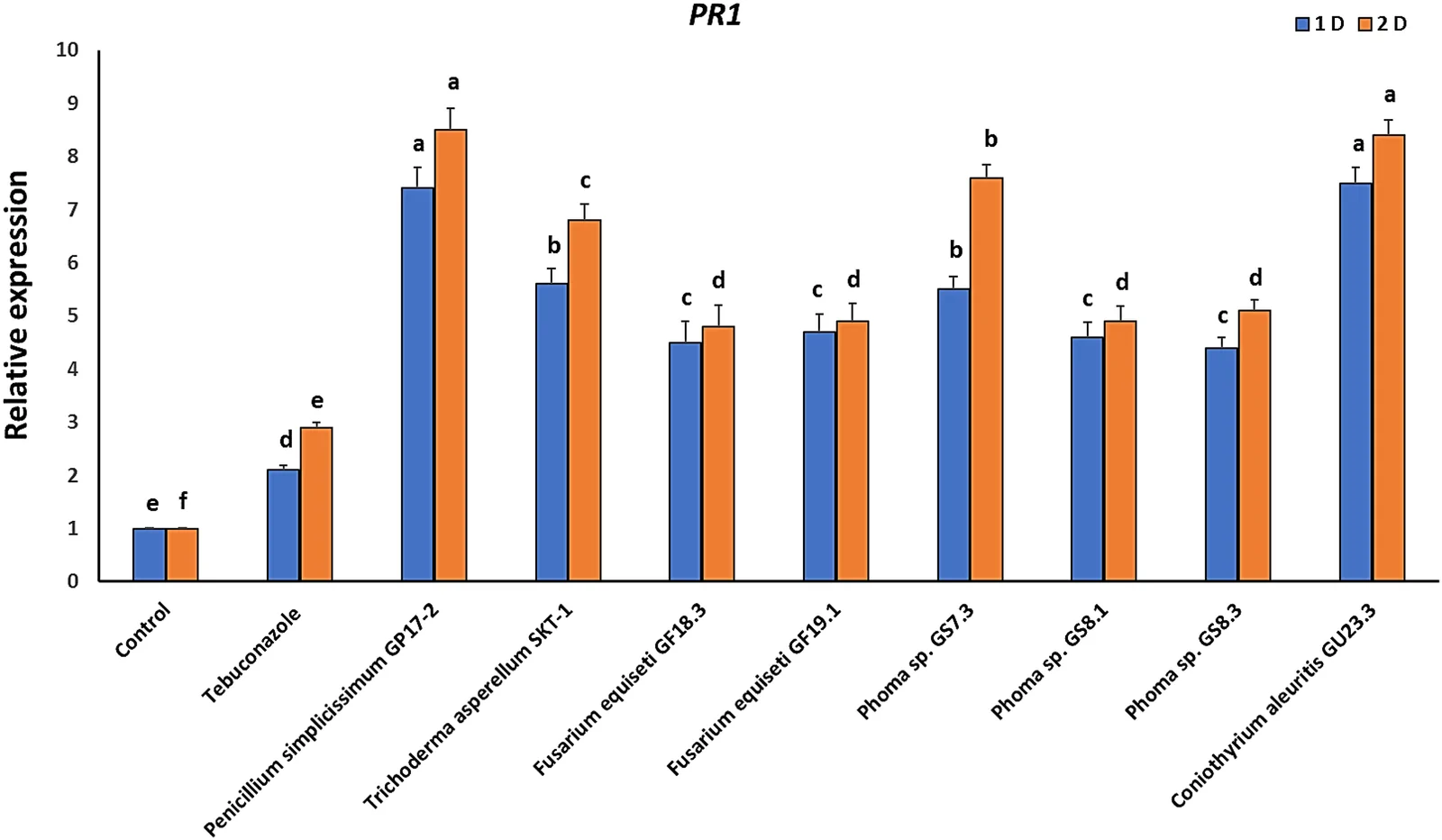
Effect of PGPF treatments on the relative expression of *PR1* gene in *B. graminis* f. sp. *tritici-*infected wheat plants at 1 and 2 days after inoculation. Values are means ± standard error (SE). Different letters indicate statistical significance between treatments at *p < 0.05*.

**Figure 6:**
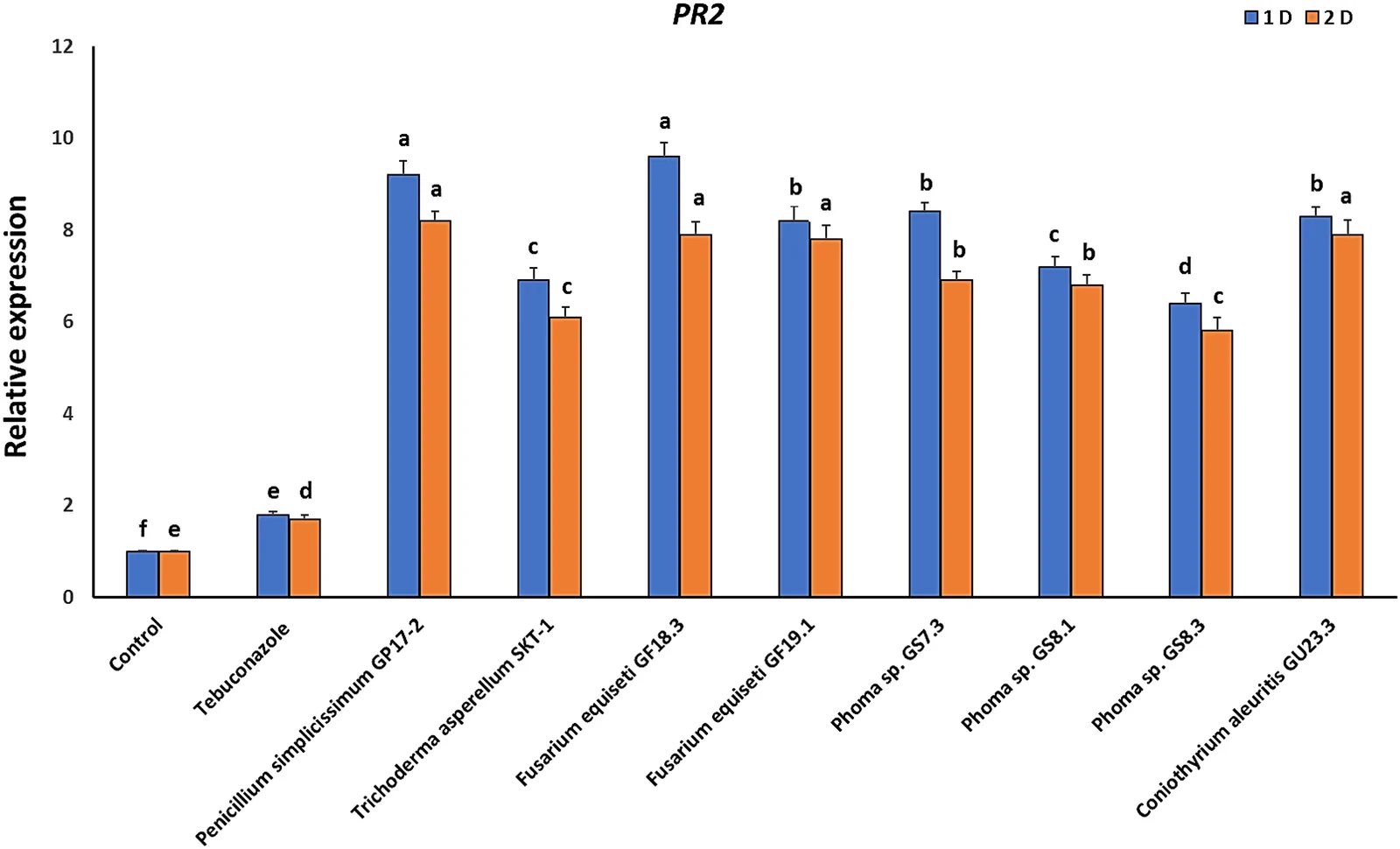
Effect of PGPF treatments on the relative expression of *PR2* gene in *B. graminis* f. sp. *tritici*-infected wheat plants at 1 and 2 days after inoculation. Values are means ± standard error (SE). Different letters indicate statistical significance between treatments at *p < 0.05*.

**Figure 7:**
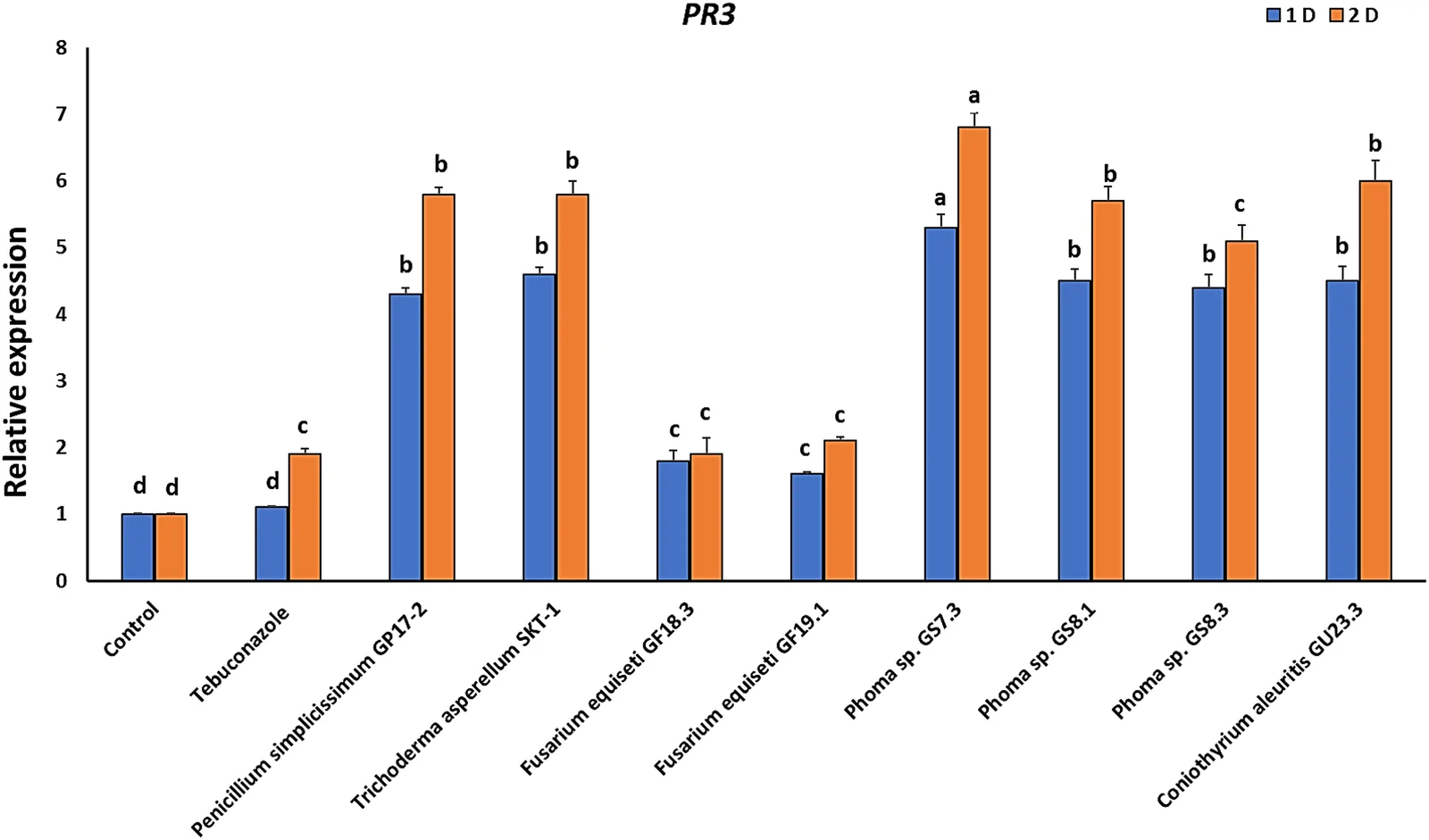
Effect of PGPF treatments on the relative expression of *PR3* gene in *B. graminis* f. sp. *tritici*-infected wheat plants at 1 and 2 days after inoculation. Values are means ± standard error (SE). Different letters indicate statistical significance between treatments at *p < 0.05*.

**Figure 8:**
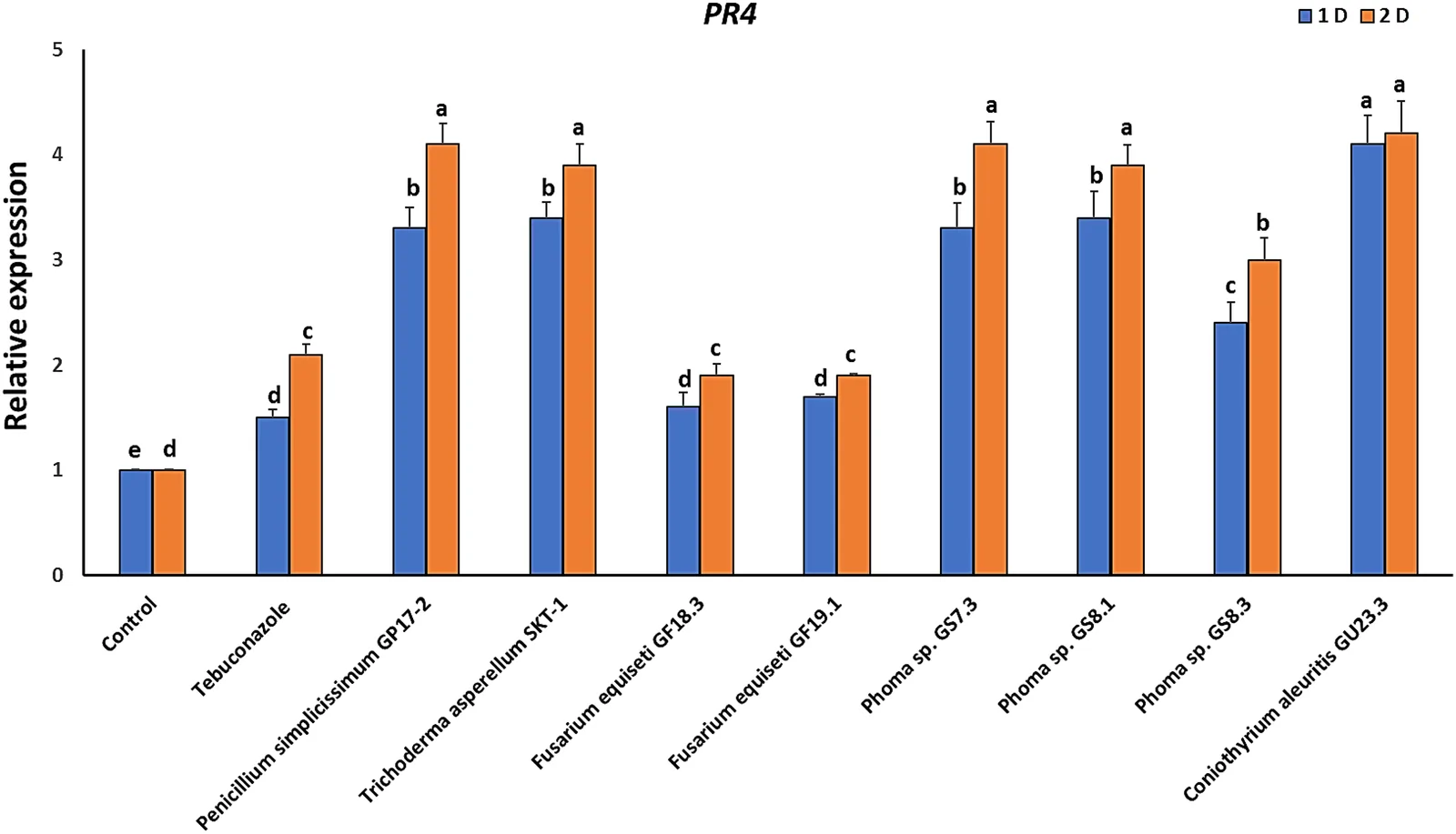
Effect of PGPF treatments on the relative expression of *PR4* gene in *B. graminis* f. sp. *tritici-*infected wheat plants at 1 and 2 days after inoculation. Values are means ± standard error (SE). Different letters indicate statistical significance between treatments at *p < 0.05*.

**Figure 9:**
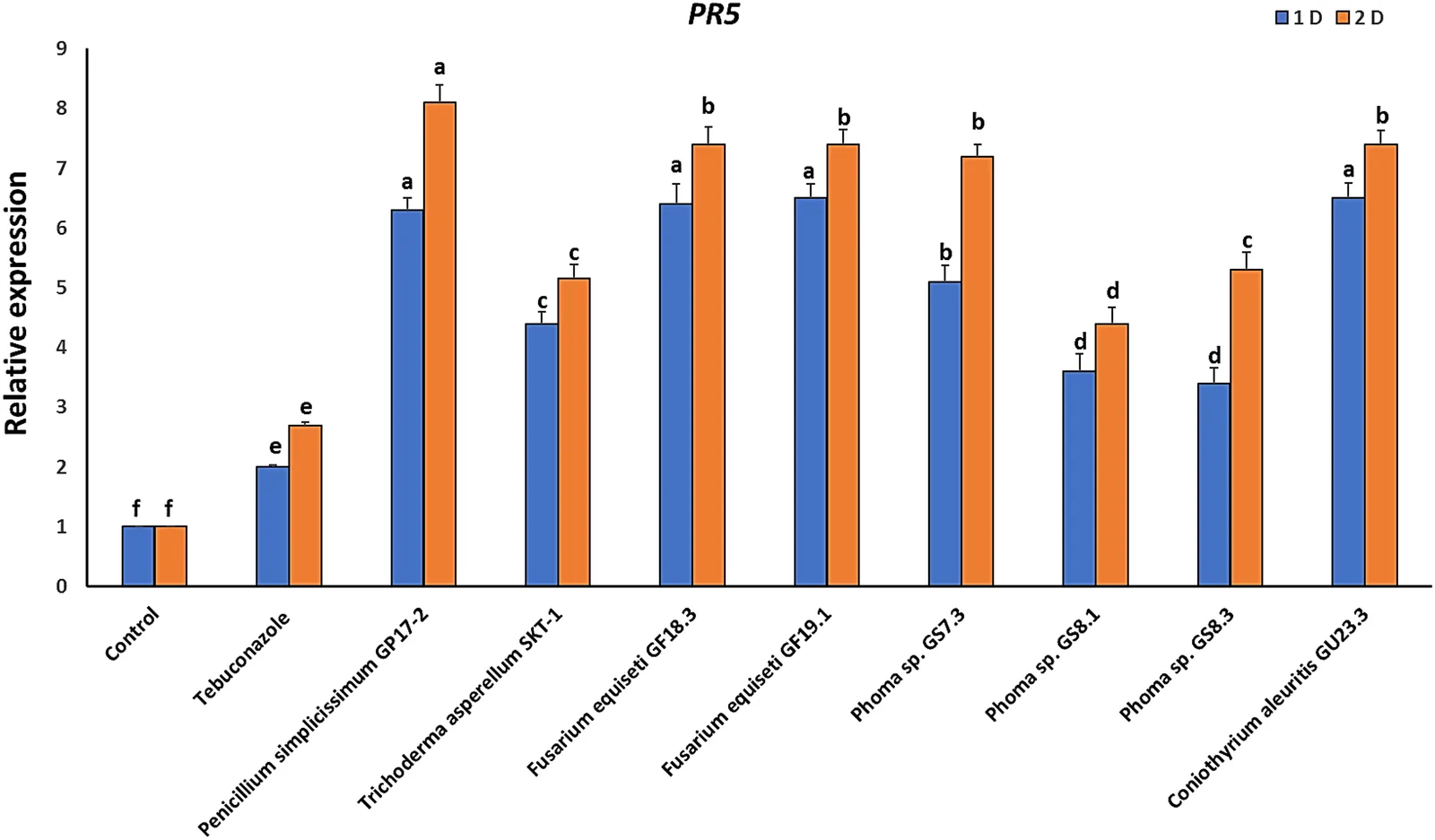
Effect of PGPF treatments on the relative expression of *PR5* gene in *B. graminis* f. sp. *tritici-*infected wheat plants at 1 and 2 days after inoculation. Values are means ± standard error (SE). Different letters indicate statistical significance between treatments at *p < 0.05*.

**Figure 10:**
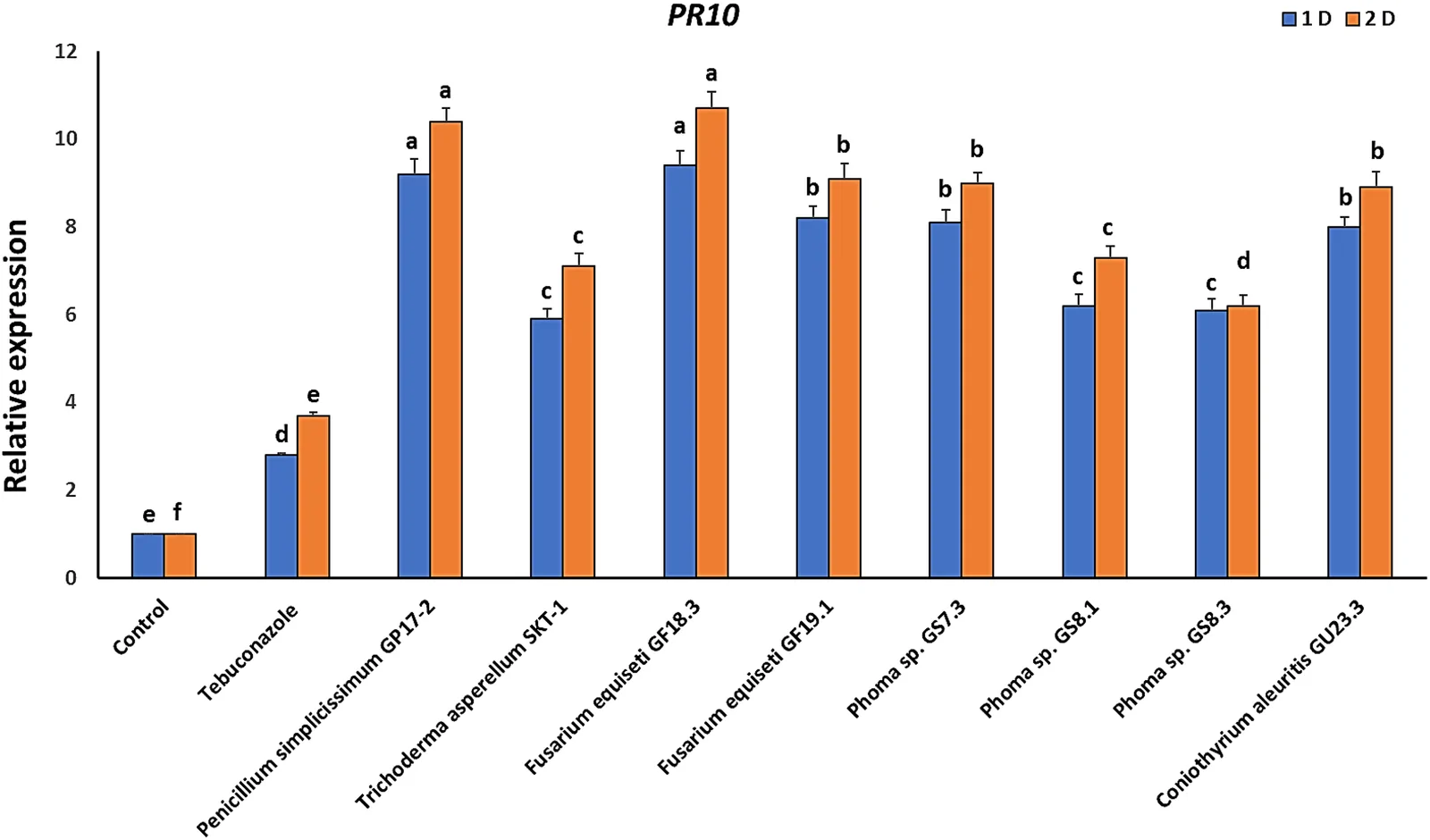
Effect of PGPF treatments on the relative expression of *PR10* gene in *B. graminis* f. sp. *tritici-*infected wheat plants at 1 and 2 days after inoculation. Values are means ± standard error (SE). Different letters indicate statistical significance between treatments at *p < 0.05*.

Induction of the genes *PR2* and *PR10* was significantly greater in the *P. simplicissimum* GP17–2 treated plants compared to *C. aleuritis* GU23.3-treated plants, and no significant differences were found in the case of *PR1* and *PR5* gene expressions at 1 DPI (**Figures 5**, **6**, **9** and **10**). *Phoma* sp. GS7.3 showed the highest upregulation of *PR3* gene (5.3-folds) (**Figure 7**). *P. simplicissimum* GP17–2 and *F. equiseti* GF18.3 treatments resulted in high stimulations of *PR2* expression without significant difference between both treatments (**Figure 6**). The gene *PR3* had the greatest expression levels in *Phoma* sp. GS7.3 (6.8-folds) at 2 DPI when compared to the control (**Figure 7**). Low expression levels of *PR4* were reported in *F. equiseti* GF18.3 and GF19.1 treatments (**Figure 8**). *PR5* was significantly stimulated to higher levels in *P. simplicissimum* GP17–2 treated plants (8.1-folds) (**Figure 9**). Both *P. simplicissimum* GP17–2 and *C. aleuritis* GU23.3 dramatically increased transcriptions of *PR1–5* without significant differences (**Figures 5**-**9**). The result is principally striking for *PR10*, which had transcription levels around 10.7 times higher in the *F. equiseti* GF18.3 treatment than the control treatment (**Figure 10**).

## Discussion

The control of wheat powdery mildew mostly depends on two commonly implemented approaches in several countries: planting resistant cultivars and using chemical fungicides (Draz et al., 2019). The powdery mildew fungus can generate new races, which may circumvent the plant’s genetic defenses. It is crucial to regularly monitor the pathogen and resistance genes in breeding programs due to the dynamic nature of the relationship between pathogen adaptation and host resistance (Abdelrhim et al., 2018). A variety of defensive mechanisms are developed by plants, allowing them to actively respond to both biotic and abiotic stresses (Choudhary et al., 2016; Shiade et al., 2024). The microbiomes of plant roots that may enhance plant health by promoting the absorption of nutrients are known as beneficial microorganisms. PGPF support the whole plant to improve and strengthen its defense against a variety of diseases via a crucial process called ISR. This study evaluates the effects of eight plant activators on plant resistance genes activation and assesses their effectiveness in controlling *B. graminis* f. sp. *tritici*. Certain plant growth-promoting microbes in the rhizosphere trigger the entire plant against a wide variety of pathogens through crucial mechanisms that enhance plant resistance (Elsharkawy and Alzahrani, 2026). Microbial inoculants (such as PGPF) could be used for development and scaling of microbial technologies for sustainable agriculture (Risoli et al., 2026). Among the evaluated PGPF, *P. simplicissimum* GP17–2 consistently displayed the best potential in decreasing disease severity. *F. equiseti* GF18–3 revealed significant suppressive effects, however *T. asperellum* SKT-1 consistently demonstrated the lowest effectiveness. The metabolites and signals produced by beneficial fungi play crucial roles in these mutually beneficial relationships, and the way these fungi interact with plants may greatly affect the long-term viability and overall quality of agroecosystems (Pérez et al., 2020). *P. simplicissimum* GP17–2 could effectively suppress the infection of cucumber mosaic virus (Elsharkawy et al., 2012). Additionally, a substantial decrease in disease symptoms and potato virus Y infection was achieved by applying *C. aleuritis* prior to PVY infection during the plant growth stage (Elsharkawy et al., 2024). The results also showed significantly increased activities of catalase and peroxidase in PGPF-treated plants. The application of the tested PGPF strains was correlated with the enhanced activities of defense enzymes in Bgt-infected wheat leaves. Wheat cells are protected from oxidative damage under stress by upregulating antioxidant enzymes such as catalase and peroxidase, which convert dangerous hydrogen peroxide into safe water and oxygen, during PGPF colonization. The enzyme peroxidase is responsible for the physical barrier that prevents pathogens from penetrating plant cell walls by cross-linking lignin and phenolics (Kumari et al., 2025).

One important strategy by which beneficial fungi in plants promote growth and stimulate defenses against various pathogens is through the ISR mechanism (Yu et al., 2022). This study assessed the potential PGPF to control Bgt and activation of wheat resistance genes. PGPF strains successfully decreased the severity of powdery mildew symptoms. Induced resistance is the key to an improved defense capacity sophisticated by wheat plants after stimulation by PGPF. PGPF-inoculated wheat plants accumulated more *PR1–5* and *PR10* relative to the control group. *Bacillus* sp. JS improved *PR2* and *PR3* regulations (Kim et al., 2015). Slowly increasing transcription of *PR9* encoding peroxidase and *PR4* encoding chitinase following volatile treatment of *Bacillus* sp. JS was observed. A lipid transmission protein gene, *PR14*, was also upregulated. The application of PGPF strains triggers systemic resistance in wheat plants (Hossain et al., 2008; Khatri et al., 2017). The average number of wilt lesions on wheat leaves decreased by 61% in plants treated with *Trichoderma koningiopsis* T-440 (El-Maraghy et al., 2020). The findings were consistent in all experiments. The PGPF strains enhanced the expression of genes related to pathogenesis *PR1* and *PR2* and produced specific proteins against the wilt pathogen (*Rhizoctonia solani*). Significant expressions of chitinase and β-1, 3-glucanase were reported in plant responses to pathogens (Fiorin et al., 2018). β-1, 3-glucan and chitin are components of fungal cell wall (Żur et al., 2013). Therefore, the fungal pathogens are thought to be sensitive to β-1, 3-glucanase and chitinase. Their ability to suppress mycelial development of the pathogens is another distinctive trait (Khatri et al., 2017). Enhanced resistance to fungal pathogens might be due to elevated expression of the *PR2* (β-1, 3-glucanase) and *PR3* (chitinase gene). A substantial reduction in symptoms of powdery mildew was detected in PGPF-treated wheat plants. The findings indicate that the use of PGPF enhanced resistance against Bgt. *PR1* gene is involved in the development of systemic resistance pathways and in improving the overall antifungal defenses. *PR1* transcription is often used as a molecular marker for the SA (salicylic acid) pathway in several plants such as barley, tomato, *Arabidopsis*, tobacco, and rice (Chinnasri et al., 2016). The importance of SA in plant defense against pathogens was reported in several research studies. SA was elevated in infected plants, and the exogenous application of SA may activate PR genes (Mathys et al., 2012). In comparison with control wheat plants, PGPF-inoculated plants stimulated the transcription of *PR1–5* and *PR10* genes. Results showed that *PR1–5* and *PR10* genes are associated with defense response pathways of Bgt infected wheat plants. PR genes produce proteins that destroy fungal structures, strengthen cell walls, and signal systemic defense. Apoptotic accumulation of *PR5* genes (thaumatin-like proteins) increases membrane permeability and disrupts fungal hyphae (Bi et al., 2025). Additional research is required to confirm strain identification, biosafety, differentiate between potential mechanisms of action, and evaluate these isolates’ effectiveness under field conditions with different ecological settings and formulation approaches.

## Conclusion

Treatment with PGPF strains resulted in less damage to the infected leaves as compared to control plants. It is possible that the reduced disease symptoms in the leaves treated with the PGPF strains are the consequence of the induced expression of defense genes. The overexpression of the defense genes was observed in PGPF treatments with fewer disease symptoms relative to the control. Among the evaluated treatments, *P. simplicissimum* GP17–2 exhibited the greatest potential in mitigating disease severity. The wheat plants treated with PGPF exhibited a highly active expression profile of the *PR1*, *PR2*, *PR3*, *PR4*, *PR5*, and *PR10* genes. *P. simplicissimum* GP17–2 and *C. aleuritis* GU23.3 might be effective in developing novel inoculants against wheat powdery mildew disease. Additionally, PGPF could be useful for the formulation of new inoculants that include a combination of multiple mechanisms of action, leading to better utilization of plant protection measures and optimized cropping systems.

## Data Availability

The original contributions presented in the study are included in the article/supplementary material, further inquiries can be directed to the corresponding author/s.

## References

[B1] Żur I. Gołębiowska G. Dubas E. Golemiec E. Matušíková I. Libantová J. . (2013). B-1, 3-glucanase and chitinase activities in winter triticales during cold hardening and subsequent infection by *Microdochium nivale*. Biologia 68, 241–248. doi: 10.2478/s11756-013-0001-0

[B2] Abdelrhim A. Abd-Alla H. M. Abdou E. S. Ismail M. E. Cowger C. (2018). Virulence of Egyptian *Blumeria graminis* f. sp. *tritici* population and response of Egyptian wheat cultivars. Plant Dis. 102, 391–397. doi: 10.1094/PDIS-07-17-0975-RE 30673514

[B3] Aebi H. (1984). Catalase *in vitro*. Methods Enzymol. 105, 121–126. doi: 10.1016/s0076-6879(84)05016-3 6727660

[B4] Alam M. A. Mandal M. S. N. Wang C. Li W. (2013). Chromosomal location and SSR markers of a powdery mildew resistance gene in chromosome wheat line N0308. Afr.J. Micro. Res. 7, 477–482. doi: 10.5897/AJMR12.1816

[B5] Asad S. Fayyaz M. Munir A. Rattu A.R. (2014). Screening of wheat commercial varieties for resistance against powdery mildew (Blumeria graminis f. sp. tritici) at Kaghan Valley, Pakistan. Pak. J. Phytopathol. 26, 7–13. Available online at: https://pjp.pakps.com/index.php/PJP/article/view/100

[B6] Annapurna K. Kumar A. Kumar L. V. Govindasamy V. Bose P. Ramadoss D. (2013). “ PGPR-induced systemic resistance (ISR) in plant disease management,” in Bacteria in Agrobiology: Disease Management. Ed. Maheshwari D. ( Springer, Berlin, Heidelberg).

[B7] Bhattacharyya P. N. Jha D. K. (2012). Plant growth-promoting rhizobacteria (PGPR) emergence in agriculture. World J. Microbiol. Biotechnol. 28, 1327–1350. doi: 10.1007/s11274-011-0979-9 22805914

[B8] Bi Q. Lu F. Wu J. Liu X. Han X. Zhao J. (2025). The control effect and induced disease resistance mechanism of *Bacillus tequilensis* on wheat powdery mildew. Biol. Control 201, 105698. doi: 10.1016/j.biocontrol.2025.10569842574925

[B9] Browder L. E. (1972). A multi-culture inoculation system for study of host-parasite relationships. Plant Dis. Rep. 56, 847–849. doi: 10.5555/19721303060

[B10] Chinnasri B. T. Borsics D. A. Christopher B. S. (2016). Induction of pathogenesis-related gene 1 (*PR-1*) by acibenzolar-s-methyl application in pineapple and its effect on reniform nematodes (*Rotylenchulus reniformis*). J. Agric. Nat. Resour. 50, 368–373. doi: 10.1016/j.anres.2016.04.00842574925

[B11] Choudhary D. K. Kasotia A. Jain S. Vaishnav A. Kumari S. Sharma K. P. . (2016). Bacterial-mediated tolerance and resistance to plants under abiotic and biotic stresses. J. Plant Growth Regul. 35, 276–300. doi: 10.1007/s00344-015-9521-x30311153

[B12] Desmond O. J. Edgar C. I. Manners J. M. Maclean D. J. Schenk P. M. Kazan K. (2006). Methyl jasmonate induced gene expression in wheat delays symptom development by the crown rot pathogen *Fusarium pseudograminearum*. Physiol. Mol. Plant Pathol. 67, 171–179. doi: 10.1016/j.pmpp.2005.12.00742574925

[B13] Draz I. S. Abdelrhim A. S. Mabrouk O. I. Esmail S. M. (2023). Potential suppression of broad-spectrum virulence of *Blumeria graminis* f. sp. *tritici* population to race- specific resistance genes in hexaploid wheat. J. Plant Pathol. 105, 1483–1496. doi: 10.1007/s42161-023-01463-530311153

[B14] Draz I. S. Esmail S. M. Abou-Zeid M. A. E. H. Essa T. A. E. M. (2019). Powdery mildew susceptibility of spring wheat cultivars as a major constraint on grain yield. Ann. Agric. Sci. 64, 39–45. doi: 10.1016/j.aoas.2019.05.00742574925

[B15] El-Maraghy S. S. Tohamy T. A. Hussein K. A. (2020). Role of plant-growth promoting fungi (PGPF) in defensive genes expression of *Triticum aestivum* against wilt disease. Rhizosphere 15, 100223. doi: 10.1016/j.rhisph.2020.10022342574925

[B16] Elsharkawy M. M. Alzahrani F. A. (2026). Antiviral activity of *Micrococcus luteus* against the infection of *bean yellow mosaic virus* in faba bean. Front. Plant Sci. 16, 1741491. doi: 10.3389/fpls.2025.1741491 41676404 PMC12886494

[B17] Elsharkawy M. M. Omara R. I. Mostafa Y. S. Alamri S. A. Hashem M. Alrumman S. A. . (2022). Mechanism of wheat leaf rust control using chitosan nanoparticles and salicylic acid. J. Fungi 8, 304. doi: 10.3390/jof8030304 35330306 PMC8950986

[B18] Elsharkawy M. M. Shimizu M. Takahashi H. Hyakumachi M. (2012). Induction of systemic resistance against Cucumber mosaic virus by *Penicillium simplicissimum* GP17-2 in *Arabidopsis* and tobacco. Plant Pathol. 61, 964–976. doi: 10.1111/ppa.2012.61.issue-5 25288946 PMC4174775

[B19] Elsharkawy M. M. Sumayli M. Alzahrani F. A. (2024). Elicitation of PVY resistance by *Coniothyrium aleuritis*. Phyton-Int J. Exp. Bot. 93, 3373–3385. doi: 10.32604/phyton.2024.058875

[B20] Esmail S. M. Al-Mutiry M. Draz I. S. Taha N. A. Shehata W. F. Marraiki N. . (2026). *Bacillus* biocontrol agents suppress wheat powdery mildew and are associated with structural alterations observed by scanning electron microscopy. Physiol. Mol. Plant Pathol. 144, 103289. doi: 10.1016/j.pmpp.2026.10328942574925

[B21] Fiorin G. L. Sanchéz-Vallet A. Thomazella D. P. do Prado P. F. V. do Nascimento L. C. Figueira A. V. . (2018). Suppression of plant immunity by fungal chitinase-like effectors. Curr. Biol. 28, 3023–3030. doi: 10.1016/j.cub.2018.07.055 30220500

[B22] Ghoniem I. R. Al-Mutiry M. Reda M. Abo-Aly M. M. Shehata W. F. Al-Maaroof E. M. . (2026). Development and validation of a QuEChERS- LC-MS/MS multiresidue method for 35 herbicides in wheat and flour: implications for food safety monitoring. Rom. Agric. Res. 43, 309–321. doi: 10.59665/rar4328

[B23] Glick B. R. (2012). Plant growth-promoting bacteria: mechanisms and applications. Life Sci. 2012, 963401. doi: 10.6064/2012/963401 24278762 PMC3820493

[B24] Hammerschmidt R. Nuckles E. M. Kuć J. (1982). Association of enhanced peroxidase activity with induced systemic resistance of cucumber to *Colletotrichum lagenarium*. Physiol. Plant Pathol. 20, 73–82. doi: 10.1016/0048-4059(82)90025-x

[B25] Hong Y. Niu J. Liu S. (2018). Impact of wheat powdery mildew on grain yield and quality and its prevention and control methods. Am. J. Agric. For. 6, 141–147. doi: 10.11648/j.ajaf.20180605.14

[B26] Hossain M. M. F. Sultana M. Kubota M. Hyakumachi M. (2008). Differential inducible defense mechanisms against bacterial speck pathogen in *Arabidopsis thaliana* by plant-growth-promoting- fungus *Penicillium* sp. GP16-2 and its cell free filtrate. Plant Soil 304, 227–239. doi: 10.1007/s11104-008-9542-330311153

[B27] Khatri D. K. Tiwari D. N. Bariya H. S. (2017). Chitinolytic efficacy and secretion of cell wall-degrading enzymes from *Trichoderma* spp. in response to phytopathological fungi. J. Chem. Technol. Biotechnol. 5, 1–8.

[B28] Kim J. S. Lee J. Lee C. Woo S. Y. Kang H. Seo S. G. . (2015). Activation of pathogenesis-related genes by the rhizobacterium, *Bacillus* sp. JS, which induces systemic resistance in tobacco plants. Plant Pathol. J. 31, 195. doi: 10.5423/ppj.nt.11.2014.0122 26060440 PMC4454002

[B29] Kumari P. Jasrotia P. Maanju S. Sareen S. Kumar D. (2025). Antioxidant enzyme responses in different wheat species infested with the corn leaf aphid, *Rhopalosiphum maidis* Fitch. Front. Plant Sci. 16, 1693782. doi: 10.3389/fpls.2025.1693782 41245462 PMC12615455

[B30] Livak K. J. Schmittgen T. D. (2001). Analysis of relative gene expression data using real-time quantitative PCR and the 2^-ΔΔCT^ method. Methods 25, 402–408. doi: 10.1006/meth.2001.1262 11846609

[B31] Lugtenberg B. Kamilova F. (2009). Plant-growth-promoting rhizobacteria. Annu. Rev. Microbiol. 63, 541–556. doi: 10.1146/annurev.micro.62.081307.162918 19575558

[B32] Mathys J. K. De Cremer P. Timmermans S. Van Kerkhove B. Lievens M. Vanhaecke B. . (2012). Genome-wide characterization of ISR induced in *Arabidopsis thaliana* by *Trichoderma hamatum* T382 against *Botrytis cinerea* infection. Front. Plant Sci. 3, 108. doi: 10.3389/fpls.2012.00108 22661981 PMC3362084

[B33] Parlevliet J. E. (1975). Partial resistance of barley to leaf rust, *Puccinia hordei*. I. Effect of cultivar and development stage on latent period. Euphytica 24, 21–27. doi: 10.1007/bf0004316130311153

[B34] Pérez L. I. Gundel P. E. Zabalgogeazcoa I. Omacini M. (2020). An ecological framework for understanding the roles of *Epichloë* endophytes on plant defenses against fungal diseases. Fungal Biol. Rev. 34, 115–125.

[B35] Rewal H. S. Jhooty J. S. (1985). Differential response of wheat varieties to systemic fungitoxicants applied *Ustilago tritici* (Pres). Indian J. Agric. Sci. 55, 548–549.

[B36] Risoli S. Turetta F. Leonardi M. Serfati A. Lorenzini G. Costanzo E. (2026). SoilTech innovations for sustainable soil and food security. Nat. Rev. Bioeng. 4, 110–111. doi: 10.1038/s44222-025-00399-y37880705

[B37] Sharma R. C. Duveiller E. (2007). Advancement toward new spot blotch resistant wheats in South Asia. Crop Sci. 47, 961–968. doi: 10.2135/cropsci2006.03.0201

[B38] Shi Q. M. Zhang X. X. Duan X. Y. Sheng B. Q. (1987). Identification of isolates of *Blumeria graminis* f. sp. *tritici*. Sci. Agric. Sin. 20, 64–70. doi: 10.1007/s10096-011-1377-9 21874606

[B39] Shiade S. R. G. Zand-Silakhoor A. Fathi A. Rahimi R. Minkina T. Rajput V. D. . (2024). Plant metabolites and signaling pathways in response to biotic and abiotic stress: Exploring bio stimulant applications. Plant Stress 12, 100454. doi: 10.1016/j.stress.2024.10045442574925

[B40] Walters D. R. Ratsep J. Havis N. D. (2013). Controlling crop diseases using induced resistance: challenges for the future. J. Exp. Bot. 64, 1263–1280. doi: 10.1093/jxb/ert026 23386685

[B41] Wang M. Xue J. Ma J. Feng X. Ying H. Xu H. (2020). *Streptomyces lydicus* M01 regulates soil microbial community and alleviates foliar disease caused by *Alternaria alternataon Cucumbers*. Front. Microbiol. 11, 942. doi: 10.3389/fmicb.2020.00942 PMC7243425 32499771

[B42] Yu Y. Gui Y. Li Z. Jiang C. Guo J. Niu D. (2022). Induced systemic resistance for improving plant immunity by beneficial microbes. Plants (Basel) 11, 386. doi: 10.3390/plants11030386 35161366 PMC8839143

[B43] Zamioudis C. Korteland J. Van Pelt J. A. van Hamersveld M. Dombrowski N. Bai Y. . (2015). Rhizobacterial volatiles and photosynthesis‐related signals coordinate MYB 72 expression in *Arabidopsis* roots during onset of induced systemic resistance and iron‐deficiency responses. Plant J. 84, 309–322. doi: 10.1111/tpj.12995 PMC5019235 26307542

